# Supplementary material for: Molecular basis of MHC I quality control in the peptide loading complex
Source: Nat Commun. 2022 Aug 10;13:4701. doi: 10.1038/s41467-022-32384-z (PMC9365787; doi:10.1038/s41467-022-32384-z)
Supplement: Supplementary file 1 — Supplementary Information [file 41467_2022_32384_MOESM1_ESM.pdf]

Supplementary information

Molecular basis of MHC I quality control in the peptide loading complex

Alexander Domnick[1,#], Christian Winter[1,#], Lukas Sušac[1], Leon Hennecke[1], Mario Hensen[2], Nicole Zitzmann[2], Simon Trowitzsch[1], Christoph Thomas[1], Robert Tampé[1*]

[1]Institute of Biochemistry, Biocenter, Goethe University Frankfurt, Max-von-Laue-Str. 9, 60438 Frankfurt am Main, Germany

[2]Oxford Glycobiology Institute, Department of Biochemistry, University of Oxford, OX1 3QU Oxford, UK

[#]Contributed equally, *To whom correspondence may be addressed

Email: tampe@em.uni-frankfurt.de

Supplementary Figure 1-7

Supplementary Table 1 and 2

Supplementary Figures

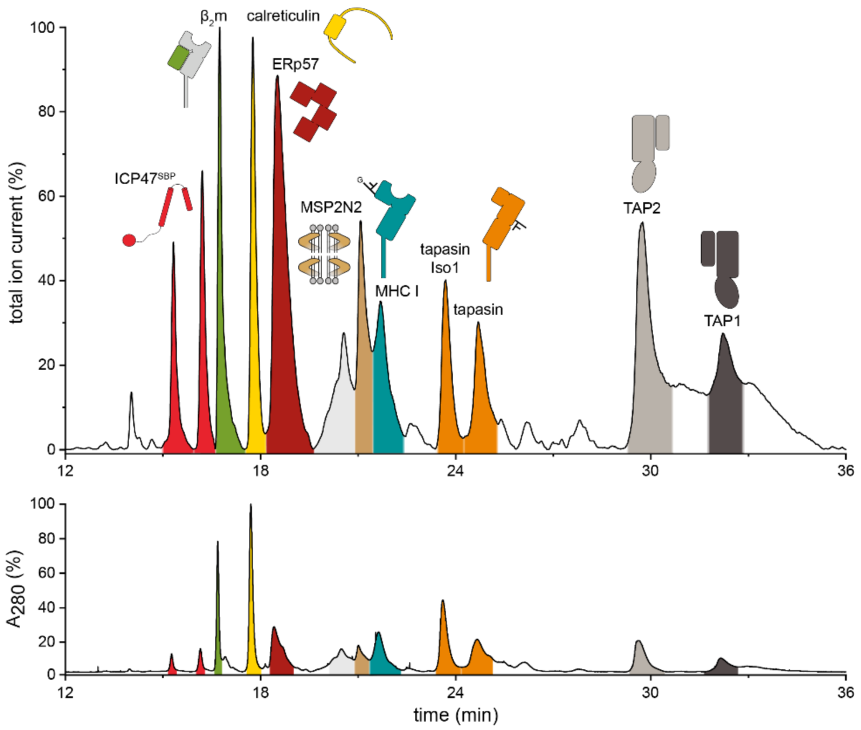

Supplementary Fig. 1 PLC subunits analyzed by LC-MS. The native, ICP47^{SBP}-bound PLC was reconstituted in large nanodiscs and subjected to LC-MS analysis. The separated PLC subunits were highlighted and assigned in their respective color in the TIC (total ion current) and A_{280}. All components of the PLC, including ICP47^{SBP} and the membrane scaffold protein MSP2N2, were successfully identified by mass spectrometry. The mass spectra and deconvoluted MS data for identified components are shown in Supplementary Fig. 2 and listed in Supplementary Table 1. ICP47^{SBP} is present in two variants, one of which contains the additional N-terminal sequence GAM. Tapasin appears in two isoforms in Raji cells.

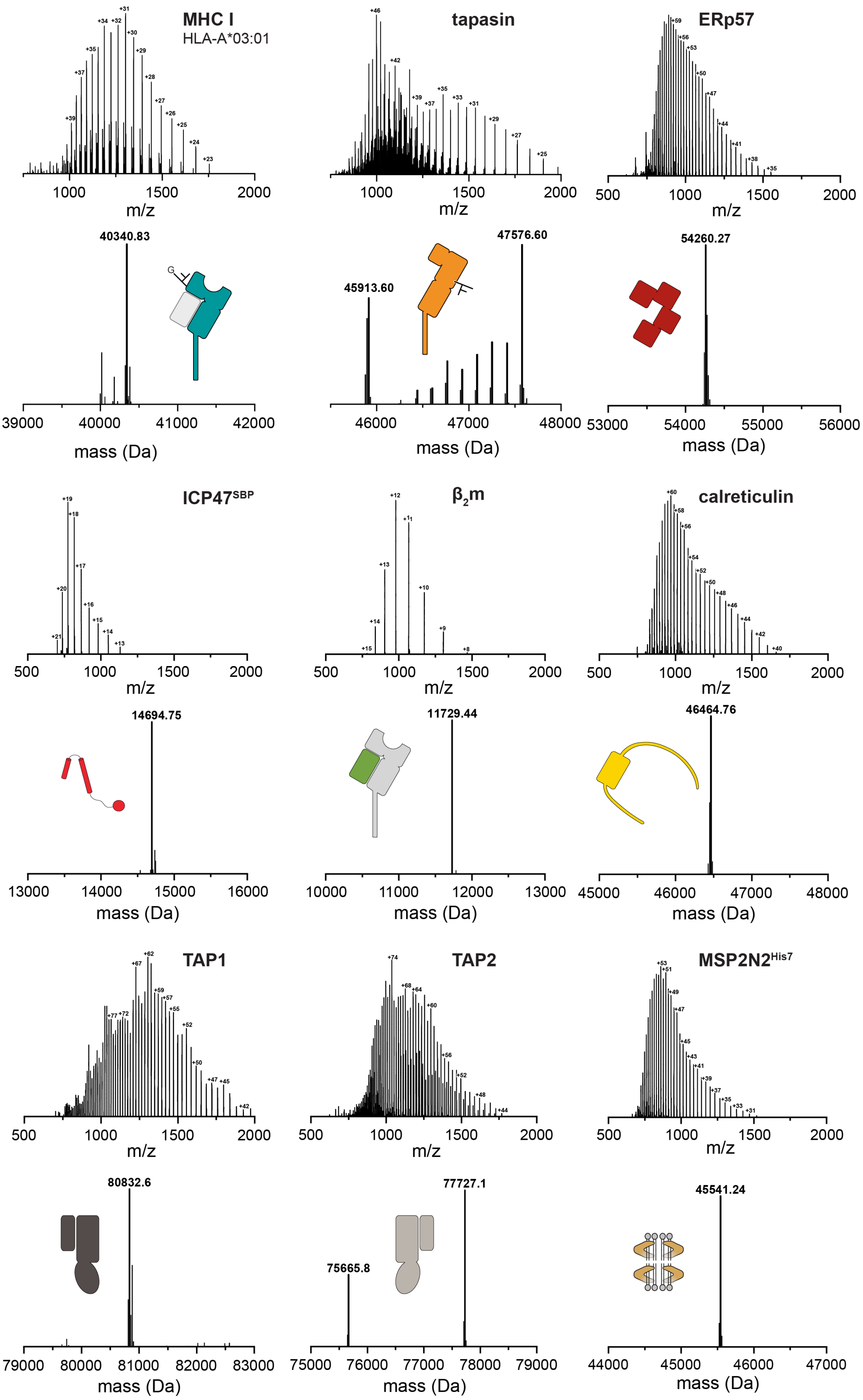

Supplementary Fig. 2 Identification of PLC subunits by intact protein LC-MS. ESI-MS and MaxEnt1 deconvoluted spectra of PLC components (Supplementary Fig. 1). The assignment of the sequences and theoretical masses of PLC components are shown in Supplementary Table 1.

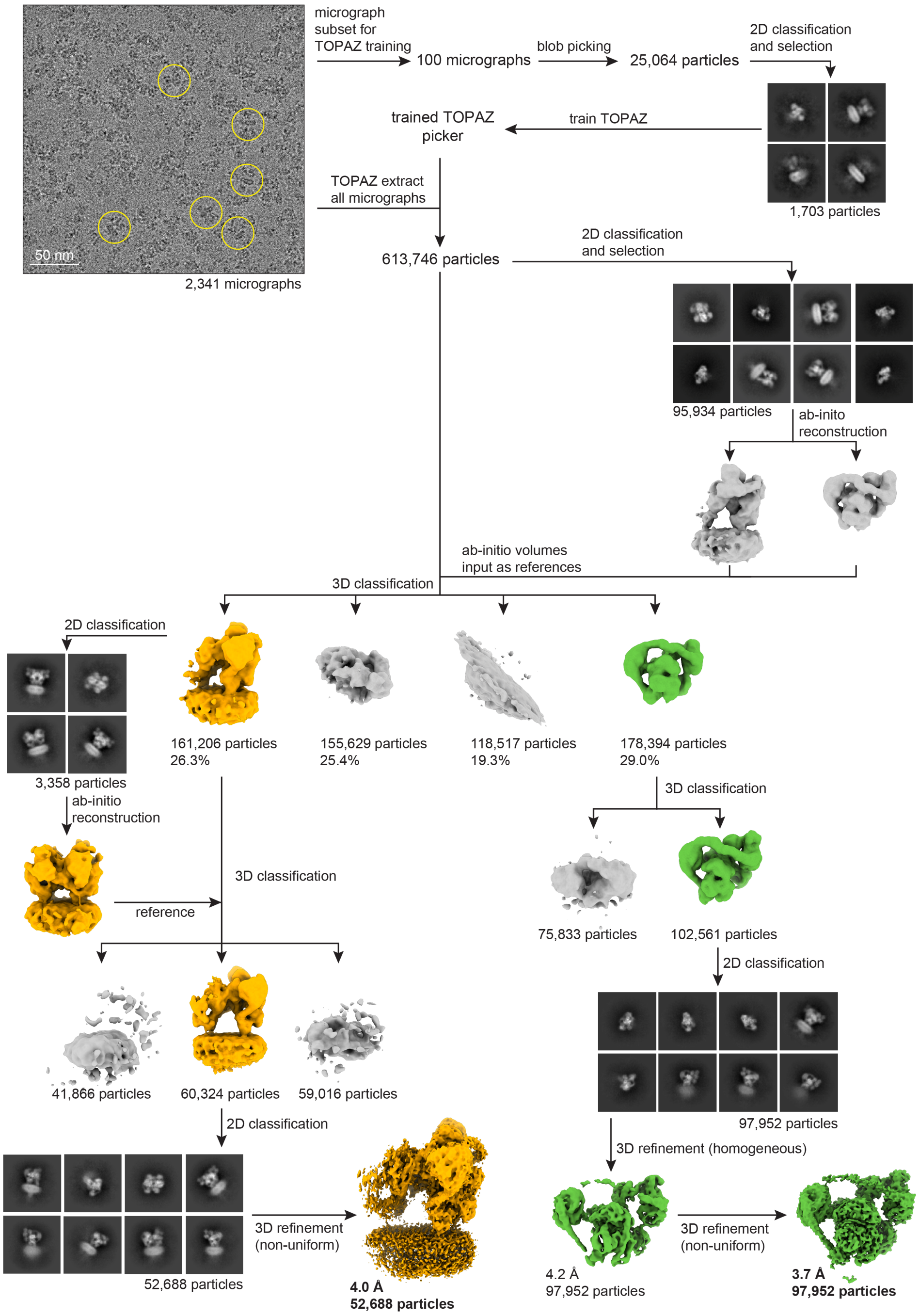

Supplementary Fig. 3 Cryo-EM data processing workflow. The full set of micrograph movies was corrected for motion and contrast transfer function in cryoSPARC. A subset of 100 micrographs was subjected to blob picking followed by 2D classification to obtain a training dataset of PLC particles for the deep picker TOPAZ. Subsequently, the trained TOPAZ instance was used to particle pick the entire set of micrographs. Initial 2D classification was then performed to select particles for ab-initio 3D reconstruction. The resulting ab-initio maps were used to classify the full dataset in 3D. 3D assemblies resembling PLC with density for the membrane region (orange, left) and a single editing module (green, right) were further sub-classified in 3D and 2D, and individually refined using homogenous and finally non-uniform refinement. All processing was performed using cryoSPARC.

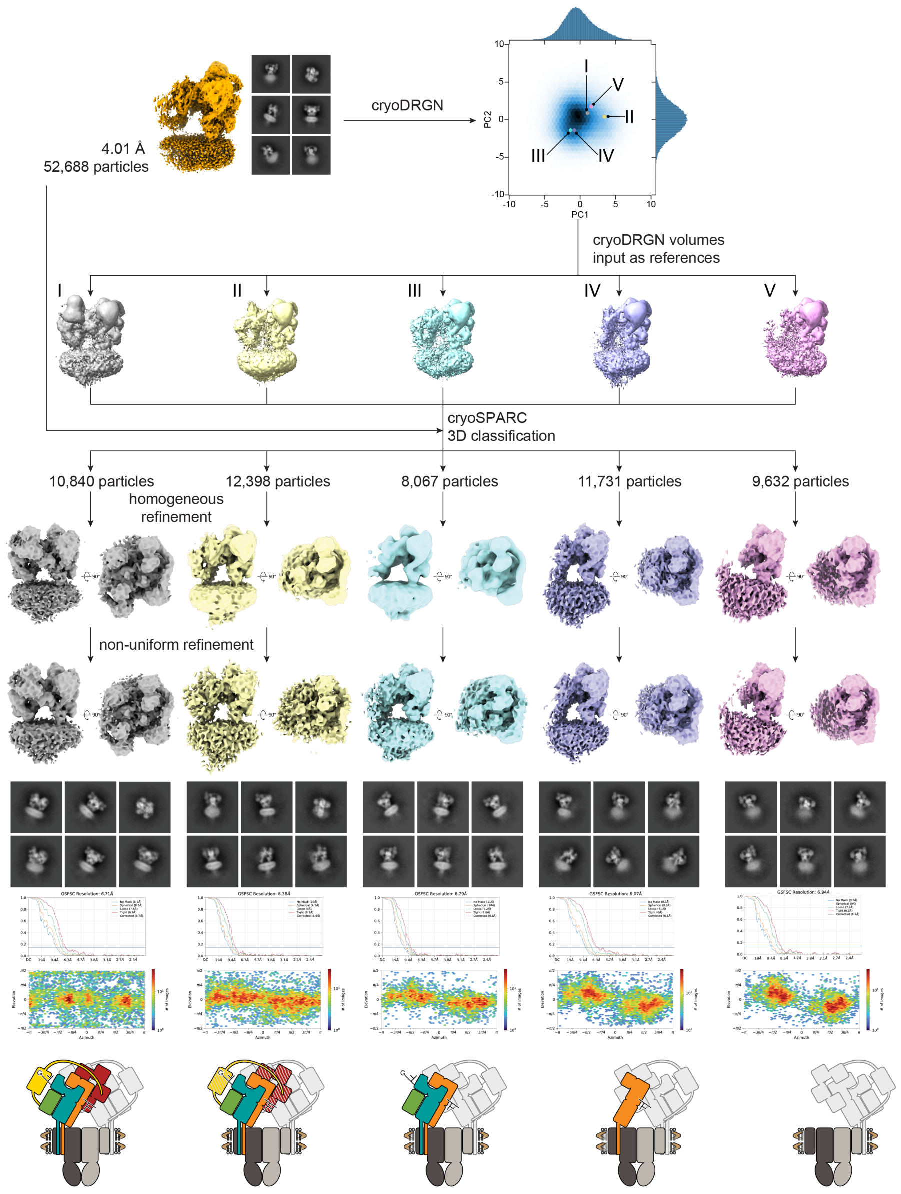

Supplementary Fig. 4 CryoDRGN analysis of different PLC assembly states. Particles of a single reconstruction from a homogeneous 3D refinement of the PLC (orange, "consensus map") were analyzed by deep learning using the neural network-based algorithm of cryoDRGN to reveal distinct PLC assemblies. Five different assembly states (I–V, colored tiles) were chosen from the principal component analysis (PCA) projection of the 8-dimensional latent space (top right) for 3D classification, followed by homogeneous and non-uniform refinement in cryoSPARC. The chosen 3D reconstructions generated by cryoDRGN showed distinct PLC assembly intermediates which could not be separated in cryoSPARC before. The fully assembled PLC (I) with two complete editing modules is shown in grey, whereas the reconstruction with a single editing module (V) is depicted in pink. Densities in yellow, blue, and purple (II – IV) represent reconstructions with intermediate assembly states of the second editing module. Depicted below each 3D map from non-uniform refinement are representative 2D class averages, Fourier shell correlation (FSC) curves of the 3D reconstructions from two independently refined half datasets, and angular assignments of particles from the final dataset with respect to the 3D structure above. Cartoons at the bottom depict the respective assembly state of the PLC by illustrating the subunits identified in each map.

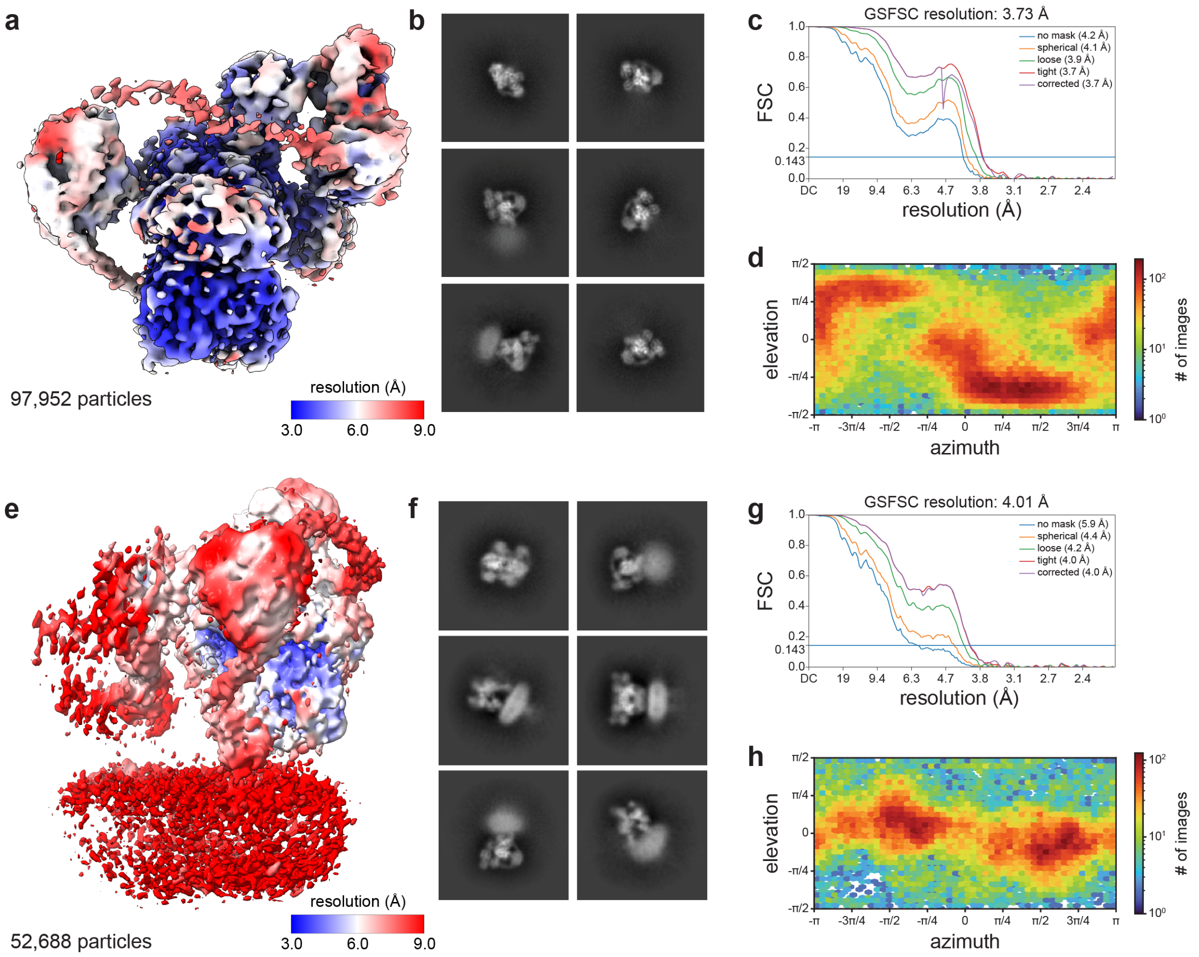

Supplementary Fig. 5 Cryo-EM analysis of the PLC reconstituted in lipid nanodiscs. a–d Single editing module. **e–f** Full PLC. **a, e** Local resolution distributions of the cryo-EM maps color-coded as indicated in the insets. **b**, **f** Representative 2D class averages from the final particle sets. Note the defined density for ER-lumenal editing module regions and blurred signal for all membrane-associated regions for the classes corresponding to the single editing module (**b**), while both editing modules are visible and the membrane region is more defined but still blurry in the classes corresponding to full PLC (**f**). **c**, **g** Fourier shell correlation (FSC) curves for the 3D reconstructions from two independently refined half datasets after non-uniform refinement in cryoSPARC. For the single editing module (**c**) and the full PLC (**g**), overall resolutions of 3.73 Å and 4.01 Å were achieved, respectively, as judged by the 0.143 threshold criterion. **d, h** Angular assignments for particles from the final datasets used for 3D reconstructions.

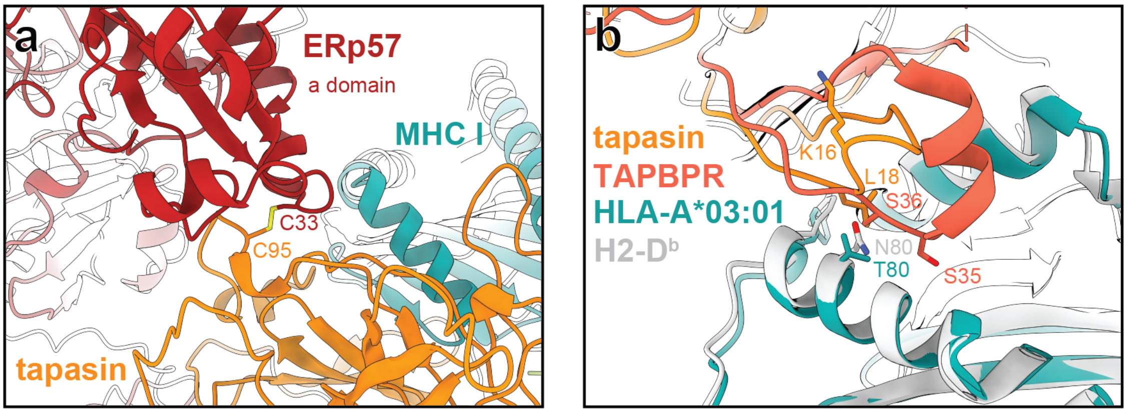

Supplementary Fig. 6 Structural features of the PLC editing. a ERp57 is linked to tapasin via an intermolecular disulfide bridge formed between Cys33 of ERp57 and Cys95 of tapasin. **b** Superposition of the tapasin editing loop in the PLC (orange/teal, PDB ID 7QPD) with the TAPBPR scoop loop in the TAPBPR-H2-Db chaperone complex (light red/light grey, PDB ID 5OPI).

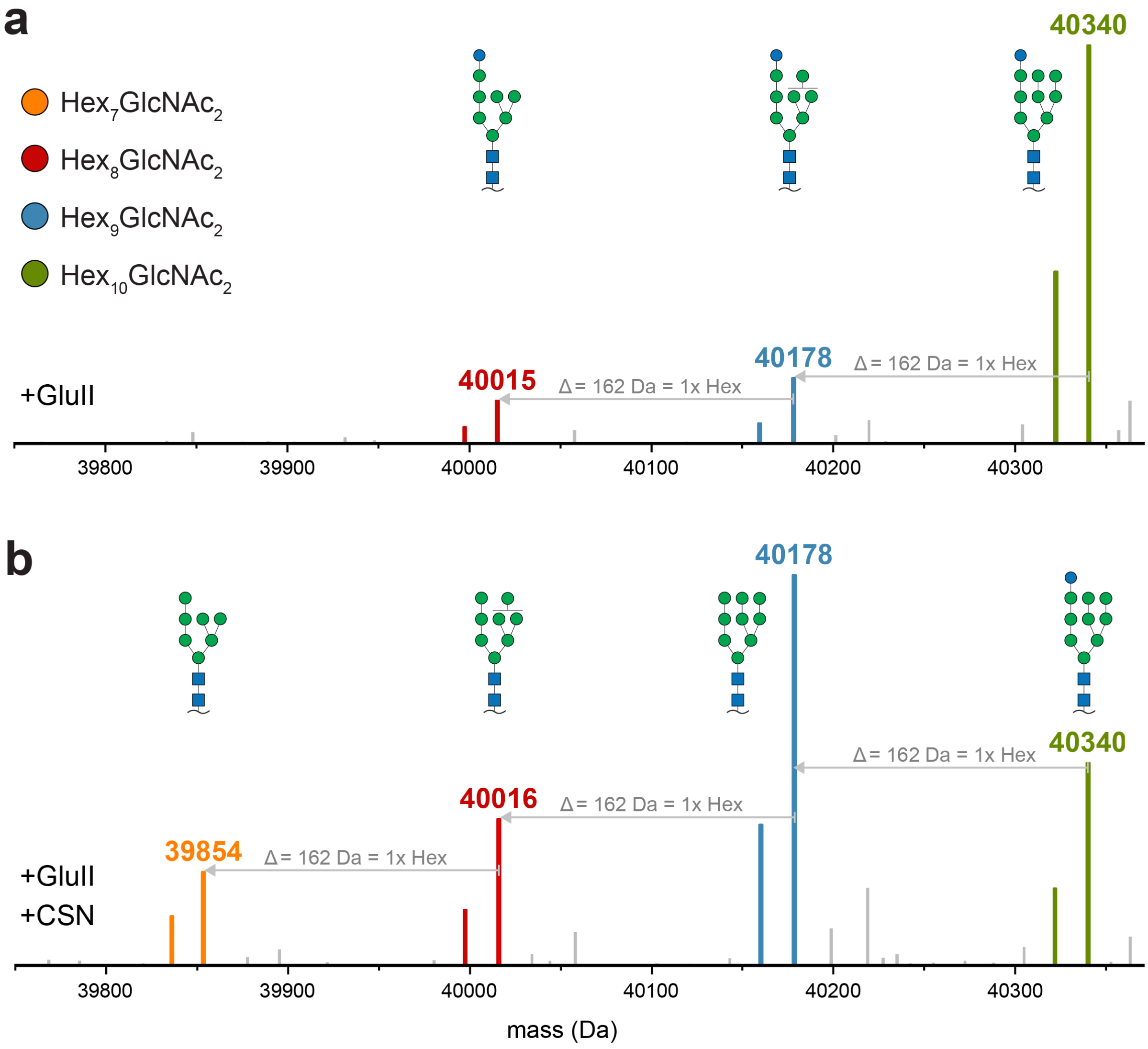

Supplementary Fig. 7 PLC-associated MHC I carries a terminal glucose in the A-branch. The minor MHC I species (Fig. 1 and Supplementary Fig. 2) carrying an N-linked glycan lack one or two mannose moieties at the B or C branches of the N-glycan tree, but still carry a terminal glucose on the A branch because GluII processing shifted the entire glycosylation pattern to lower molecular weight (MW). Deconvoluted MS spectra of HLA-A*03:01 are shown. **a** In the absence of peptides, no processing by GluII is detectable. The major HLA-A*03:01 fraction carries the $Glc_1Man_9GlcNAc_2$ glycan. A minor fraction of HLA-A*03:01 species harbor glycans with only nine (blue) or eight hexoses (red). **b** In the presence of high-affinity peptides, the pattern is shifted to lower MW and a new species with seven hexoses (orange) appears. The lower intensity mass peaks, lacking 18 Da in comparison to the labeled mass peaks, are a common artifact of rapid thermal decomposition inside the ESI-MS source,[46] which appeared to be more pronounced in glycoproteins.

Supplementary Table 1 | Subunits of the PLC identified by LC-MS

Component (reference)	AA Sequence (mature protein)	M_{cal} (Da)	M_{obs} (Da)
**β$_2$m (Uniprot: P61769)**	IQRTPKIQVY SRHPAENGKS NFLNCYVSGF HPSDIEVDLL<br>KNGERIEKVE HSDLSFSKDW SFYLLYYTEF TPTEKDEYAC<br>RVNHVTLSQP KIVKWDRDM	11729.01	11729.44
**calreticulin (Uniprot: P27797)**	EPAVYFKEQF LDGDGWTSRW IESKHKSDFG KFVLSSGKFY<br>GDEEKDKGLQ TSQDARFYAL SASFEPFSNK GQTLVVQFTV<br>KHEQNIDCGG GYVKLFPNSL DQTDMHGDSE YNIMFGPDIC<br>GPGTKKVHVI FNYKGKNVLI NKDIRCKDDE FTHLYTLIVR<br>PDNTYEVKID NSQVESGSLE DDWDFLPPKK IKDPDASKPE<br>DWDERAKIDD PTDSKPEDWD KPEHIPDPDA KKPEDWDEEM<br>DGEWEPPVIQ NPEYKGEWKP RQIDNPDYKG TWIHPEIDNP<br>EYSPDPSIYA YDNFGVLGLD LWQVKSGTIF DNFLITNDEA<br>YAEEFGNETW GVTKAAEKQM KDKQDEEQRL KEEEEDKKRK<br>EEEEAEDKED DEDKDEDEED EEDKEEDEEE DVPGQAKDEL	46463.77	46464.76
**ERp57 (Uniprot: P30101)**	SDVLELTDDN FESRISDTGS AGLMLVEFFA PWCGHCKRLA<br>PEYEAAATRL KGIVPLAKVD CTANTNTCNK YGVSGYPTLK<br>IFRDGEEAGA YDGPRTADGI VSHLKKQAGP ASVPLRTEEE<br>FKKFISDKDA SIVGFFDDSF SEAHSEFLKA ASNLRDNYRF<br>AHTNVESLVN EYDDNGEGII LFRPSHLTNK FEDKTVAYTE<br>QKMTSGKIKK FIQENIFGIC PHMTEDNKDL IQGKDLLIAY<br>YDVDYEKNAK GSNYWRNRVM MVAKKFLDAG HKLNFAVASR<br>KTFSHELSDF GLESTAGEIP VVAIRTAKGE KFVMQEEFSR<br>DGKALERFLQ DYFDGNLKRY LKSEPIPESN DGPVKVVVAE<br>NFDEIVNNEN KDVLIEFYAP WCGHCKNLEP KYKELGEKLS<br>KDPNIVIAKM DATANDVPSP YEVRGFPTIY FSPANKKLNP<br>KKYEGGRELS DFISYLQREA TNPPVIQEEK PKKKKKAQED L	54260.53	54260.27
**HLA-A*03:01 (NCBI: NP_002107.3)**	GSHSMRYFFT SVSRPGRGEP RFIAVGYVDD TQFVRFDSDA<br>ASQRMEPRAP WIEQEGPEYW DQETRNVKAQ SQTDRVDLGT<br>LRGYYNQSEA GSHTIQIMYG CDVGSDGRFL RGYRQDAYDG<br>KDYIALNEDL RSWTAADMAA QITKRKWEAA HEAEQLRAYL<br>DGTCVEWLRR YLENGKETLQ RTDPPKTHMT HHPISDHEAT<br>LRCWALGFYP AEITLTWQRD GEDQTQDTEL VETRPAGDGT<br>FQKWAAVVVP SGEEQRYTCH VQHEGLPKPL TLRWELSSQP<br>TIPIVGIIAG LVLLGAVITG AVVAAVMWRR KSSDRKGGSY<br>TQAASSDSAQ GSDVSLTACK V	38312.12	38313.27
**tapasin Iso1 (Uniprot: O15533)**	GPAVIECWFV EDASGKGLAK RPGALLLRQG PGEPPPRPDL<br>DPELYLSVHD PAGALQAAFR RYPRGAPAPH CEMSRFVPLP<br>ASAKWASGLT PAQNCPRALD GAWLMVSISS PVLSLSSLLR<br>PQPEPQQEPV LITMATVVLT VLTHTPAPRV RLGQDALLDL<br>SFAYMPPTSE AASSLAPGPP PFGLEWRRQH LGKGHLLLAA<br>TPGLNGQMPA AQEGAVAFAA WDDDEPWGPW TGNGTFWLPR<br>VQPFQEGTYL ATIHLPYLQG QVTLELAVYK PPKVSLMPAT<br>LARAAPGEAP PELLCLVSHF YPSGGLEVEW ELRGGPGGRS<br>QKAEGQRWLS ALRHHSDGSV SLSGHLQPPP VTTEQHGARY<br>ACRIHHPSLP ASGRSAEVTL EVAGLSGPSL EDSVGLFLSA<br>FLLLGLFKAL GWAAVYLSTC KDSKKKAE	45711.82	45710.70
**TAP2 – Iso1 A665T[1] (NCBI: NP_000535.3)**	MRLPDLRPWT SLLLVDAALL WLLQGPLGTL LPQGLPGLWL<br>EGTLRLGGLW GLLKLRGLLG FVGTLLLPLC LATPLTVSLR<br>ALVAGASRAP PARVASAPWS WLLVGYGAAG LSWSLWAVLS<br>PPGAQEKEQD QVNNKVLMWR LLKLSRPDLP LLVAAFFFLV<br>LAVLGETLIP HYSGRVIDIL GGDFDPHAFA SAIFFMCLFS<br>FGSSLSAGCR GGCFTYTMSR INLRIREQLF SSLLRQDLGF<br>FQETKTGELN SRLSSDTTLM SNWLPLNANV LLRSLVKVVG<br>LYGFMLSISP RLTLLSLLHM PFTIAAEKVY NTRHQEVLRE<br>IQDAVARAGQ VVREAVGGLQ TVRSFGAEEH EVCRYKEALE<br>QCRQLYWRRD LERALYLLVR RVLHLGVQML MLSCGLQQMQ<br>DGELTQGSLL SFMIYQESVG SYVQTLVYIY GDMLSNVGAA<br>EKVFSYMDRQ PNLPSPGTLA PTTLQGVVKF QDVSFAYPNR	77724.23	77727.10

	PDRPVLKGLT FTLRPGEVTA LVGPNGSGKS TVAALLQNLY<br>QPTGGQVLLD EKPISQYEHC YLHSQVVSVG QEPVLFSGSV<br>RNNIAYGLQS CEDDKVMAAA QAAHADDFIQ EMEHGIYTDV<br>GEKGSQLAAG QKQRLAIARA LVRDPRVLIL DEATSALDVQ<br>CEQALQDWNS RGDRTVLVIA HRLQTVQRAH QILVLQEGKL<br>QKLAQLQEGQ DLYSRLVQQR LMD		
**TAP2 – Iso3 (NCBI: NP_ 001276972.1)**	MRLPDLRPWT SLLLVDAALL WLLQGPLGTL LPQGLPGLWL<br>EGTLRLGGLW GLLKLRGLLG FVGTLLLPLC LATPLTVSLR<br>ALVAGASRAP PARVASAPWS WLLVGYGAAG LSWSLWAVLS<br>PPGAQEKEQD QVNNKVLMWR LLKLSRPDLP LLVAAFFFLV<br>LAVLGETLIP HYSGRVIDIL GGDFDPHAFA SAIFFMCLFS<br>FGSSLSAGCR GGCFTYTMSR INLRIREQLF SSLLRQDLGF<br>FQETKTGELN SRLSSDTTLM SNWLPLNANV LLRSLVKVVG<br>LYGFMLSISP RLTLLSLLHM PFTIAAEKVY NTRHQEVLRE<br>IQDAVARAGQ VVREAVGGLQ TVRSFGAEEH EVCRYKEALE<br>QCRQLYWRRD LERALYLLVR RVLHLGVQML MLSCGLQQMQ<br>DGELTQGSLL SFMIYQESVG SYVQTLVYIY GDMLSNVGAA<br>EKVFSYMDRQ PNLPSPGTLA PTTLQGVVKF QDVSFAYPNR<br>PDRPVLKGLT FTLRPGEVTA LVGPNGSGKS TVAALLQNLY<br>QPTGGQVLLD EKPISQYEHC YLHSQVVSVG QEPVLFSGSV<br>RNNIAYGLQS CEDDKVMAAA QAAHADDFIQ EMEHGIYTDV<br>GEKGSQLAAG QKQRLAIARA LVRDPRVLIL DEATSALDVQ<br>CEQALQDWNS RGDRTVLVIA HRLQTVQRAH QILVLQEGKL<br>QKLAQL	75662.95	75665.80
**TAP1 - Iso1 (NCBI: NP_ 000584)**	ASSRCPAPRG CRCLPGASLA WLGTVLLLLA DWVLLRTALP<br>RIFSLLVPTA LPLLRVWAVG LSRWAVLWLG ACGVLRATVG<br>SKSENAGAQG WLAALKPLAA ALGLALPGLA LFRELISWGA<br>PGSADSTRLL HWGSHPTAFV VSYAAALPAA ALWHKLGSLW<br>VPGGQGGSGN PVRRLLGCLG SETRRLSLFL VLVVLSSLGE<br>MAIPFFTGRL TDWILQDGSA DTFTRNLTLM SILTIASAVL<br>EFVGDGIYNN TMGHVHSHLQ GEVFGAVLRQ ETEFFQQNQT<br>GNIMSRVTED TSTLSDSLSE NLSLFLWYLV RGLCLLGIML<br>WGSVSLTMVT LITLPLLFLL PKKVGKWYQL LEVQVRESLA<br>KSSQVAIEAL SAMPTVRSFA NEEGEAQKFR EKLQEIKTLN<br>QKEAVAYAVN SWTTSISGML LKVGILYIGG QLVTSGAVSS<br>GNLVTFVLYQ MQFTQAVEVL LSIYPRVQKA VGSSEKIFEY<br>LDRTPRCPPS GLLTPLHLEG LVQFQDVSFA YPNRPDVLVL<br>QGLTFTLRPG EVTALVGPNG SGKSTVAALL QNLYQPTGGQ<br>LLLDGKPLPQ YEHRYLHRQV AAVGQEPQVF GRSLQENIAY<br>GLTQKPTMEE ITAAAVKSGA HSFISGLPQG YDTEVDEAGS<br>QLSGGQRQAV ALARALIRKP CVLILDDATS ALDANSQLQV<br>EQLLYESPER YSRSVLLITQ HLSLVEQADH ILFLEGGAIR<br>EGGTHQQLME KKGCYWAMVQ APADAPE	80832.50	80832.60
**ICP47^{SBP}**<br>**^{GAM}ICP47^{SBP}**	GAMASWALEM ADTFLDNMRV GPRTYADVRD EINKRGREDR<br>EAARTAVHDP ERPLLRSPGL LPEIAPNASL GVAHRRTGGT<br>VTDSPRNPVT RGSGGGSGGG SMDEKTTGWR GGHVVEGLAG<br>ELEQLRARLE HHPQGQREP	14953.39<br>14694.07	14953.80<br>14694.75
**MSP2N2**	GHHHHHHHDY DIPTTENLYF QGSTFSKLRE QLGPVTQEFW<br>DNLEKETEGL RQEMSKDLEE VKAKVQPYLD DFQKKWQEEM<br>ELYRQKVEPL RAELQEGARQ KLHELQEKLS PLGEEMRDRA<br>RAHVDALRTH LAPYSDELRQ RLAARLEALK ENGGARLAEY<br>HAKATEHLST LSEKAKPALE DLRQGLLPVL ESFKVSFLSA<br>LEEYTKKLNT QGTPVTQEFW DNLEKETEGL RQEMSKDLEE<br>VKAKVQPYLD DFQKKWQEEM ELYRQKVEPL RAELQEGARQ<br>KLHELQEKLS PLGEEMRDRA RAHVDALRTH LAPYSDELRQ<br>RLAARLEALK ENGGARLAEY HAKATEHLST LSEKAKPALE<br>DLRQGLLPVL ESFKVSFLSA LEEYTKKLNT Q	45540.70	45541.24

Supplementary Table 2 Cryo-EM data collection, refinement, and validation statistics

	PLC Editing Module<br>Calreticulin-tapasin-ERp57-MHC I hc-β_2m<br>(EMDB-14119)<br>(PDB ID 7QPD)
**Data collection and processing**	
Microscope	TITAN-Krios
Camera	Gatan K2 Summit
Magnification	130,000
Voltage (kV)	300
Electron exposure (e–/Å^2)	68
Defocus range (μm)	-1.0 to -2.5
Pixel size (Å)	1.05
Symmetry imposed	C1
Initial particle images (no.)	613,746
Final particle images (no.)	97,952
Map resolution (Å)	3.73
FSC threshold	0.143
**Refinement**	
Map sharpening *B* factor (Å^2)	-81.1
Model composition	
Non-hydrogen atoms	11,521
Protein residues	1544
Carbohydrate	10
B factors (Å^2)	
Protein	63.89
Carbohydrate	87.35
R.m.s. deviations	
Bond lengths (Å)	0.0047
Bond angles (°)	0.78
Validation	
MolProbity score	1.85
Clashscore	9.50
Poor rotamers (%)	0.09
Carbohydrates[1]	
Stereochemical problems	0
Unphysical puckering amplitude	0
In unlikely ring conformation	0
Ramachandran plot	
Favored (%)	95.01
Allowed (%)	4.92
Disallowed (%)	0.07

[1]As reported by the Privateer software package
