# Supplementary material for: Molecular basis of MHC I quality control in the peptide loading complex
Source: Nat Commun. 2022 Aug 10;13:4701. doi: 10.1038/s41467-022-32384-z (PMC9365787; doi:10.1038/s41467-022-32384-z)
Supplement: Supplementary file 3 — Reporting Summary [file 41467_2022_32384_MOESM3_ESM.pdf]

nature portfolio

Corresponding author(s): Robert Tampé

Last updated by author(s): July 11, 2022

Reporting Summary

Nature Portfolio wishes to improve the reproducibility of the work that we publish. This form provides structure for consistency and transparency in reporting. For further information on Nature Portfolio policies, see our Editorial Policies and the Editorial Policy Checklist.

Statistics

For all statistical analyses, confirm that the following items are present in the figure legend, table legend, main text, or Methods section.

n/a	Confirmed	
☐	☒	The exact sample size (*n*) for each experimental group/condition, given as a discrete number and unit of measurement
☒	☐	A statement on whether measurements were taken from distinct samples or whether the same sample was measured repeatedly
☒	☐	The statistical test(s) used AND whether they are one- or two-sided<br>*Only common tests should be described solely by name; describe more complex techniques in the Methods section.*
☒	☐	A description of all covariates tested
☒	☐	A description of any assumptions or corrections, such as tests of normality and adjustment for multiple comparisons
☐	☒	A full description of the statistical parameters including central tendency (e.g. means) or other basic estimates (e.g. regression coefficient) AND variation (e.g. standard deviation) or associated estimates of uncertainty (e.g. confidence intervals)
☒	☐	For null hypothesis testing, the test statistic (e.g. F, t, r) with confidence intervals, effect sizes, degrees of freedom and P value noted<br>*Give P values as exact values whenever suitable.*
☒	☐	For Bayesian analysis, information on the choice of priors and Markov chain Monte Carlo settings
☒	☐	For hierarchical and complex designs, identification of the appropriate level for tests and full reporting of outcomes
☒	☐	Estimates of effect sizes (e.g. Cohen's d, Pearson's r), indicating how they were calculated

Our web collection on statistics for biologists contains articles on many of the points above.

Software and code

Policy information about availability of computer code

Data collection	cryo-EM: SerialEM V 3.6<br>LC-MS: Waters BioAccord System<br>Peitide binding: BMG Labtech CLARIOstar V 5.20 R5<br>SEC: LabSolutions V 5.81 SP1, Shimadzu Corporation
Data analysis	cryo-EM: CryoSPARC v2.15 - 3.2 (ref. 35); Topaz (ref. 36,37); CryoDRGN (ref. 38); ISOLDE (ref. 39); COOT (ref. 40); Phenix (ref. 41); Privateer (ref. 42); CCP4 software suite (ref. 43), ChimeraX V 1.1<br>MHC I peptide affinity: NetMHC 4.1 (ref. 34)<br>LC-MS: Waters UNIFY 1.9.4.053<br>general: OriginLab OriginPro 2020

For manuscripts utilizing custom algorithms or software that are central to the research but not yet described in published literature, software must be made available to editors and reviewers. We strongly encourage code deposition in a community repository (e.g. GitHub). See the Nature Portfolio guidelines for submitting code & software for further information.

Data

Policy information about availability of data

All manuscripts must include a data availability statement. This statement should provide the following information, where applicable:

- Accession codes, unique identifiers, or web links for publicly available datasets
- A description of any restrictions on data availability
- For clinical datasets or third party data, please ensure that the statement adheres to our policy

LC-MS raw data were submitted to the open-access repository Zenodo with the Digital Object Identifier (DOI) 10.5281/zenodo.5793891. The cryo-EM density maps and the corresponding model were deposited in the Electron Microscopy Data Bank under accession numbers EMD-14119 and PDB ID 7QPD. Source data are provided with this paper.

Field-specific reporting

Please select the one below that is the best fit for your research. If you are not sure, read the appropriate sections before making your selection.

☒ Life sciences ☐ Behavioural & social sciences ☐ Ecological, evolutionary & environmental sciences

For a reference copy of the document with all sections, see nature.com/documents/nr-reporting-summary-flat.pdf

Life sciences study design

All studies must disclose on these points even when the disclosure is negative.

Sample size	Immunoblotting: All Immunoblots were performed at least two times as stated in the figure legends.<br><br>cryo-EM: 3D reconstructions were calculated from 2,341 images (613,746 particles).<br><br>Intact protein LC-MS:<br>PLC component MS confirmation: Intact protein Mass of PLC components were confirmed in every PLC analysis in the dataset (n=12).<br>LC-MS deglucosylation assay: Each sample was prepared in biological duplicates.
Data exclusions	Biochemistry: No data were excluded.<br><br>Intact protein LC-MS: Initial screening experiments for ideal chromatography and mass spectrometry conditions were not included in this dataset.<br><br>cryo-EM: particles that do not belong to the class of interest or have poor qualities were excluded after 2D and 3D classification. This standard procedure is required to obtain high resolution cryo-EM structures for proteins and assemblies of interest.
Replication	All attempts stated in the figure legends were performed successfully
Randomization	Since this study did not involve research on living organisms, no confounding factors requiring randomization were expected. No randomization has been performed.
Blinding	Since this study did not involve research on living organisms, no confounding factors requiring blinding were expected. No blinding has been performed.

Reporting for specific materials, systems and methods

We require information from authors about some types of materials, experimental systems and methods used in many studies. Here, indicate whether each material, system or method listed is relevant to your study. If you are not sure if a list item applies to your research, read the appropriate section before selecting a response.

Materials & experimental systems

n/a	Involved in the study	
☐	☒	Antibodies
☐	☒	Eukaryotic cell lines
☒	☐	Palaeontology and archaeology
☒	☐	Animals and other organisms
☒	☐	Human research participants
☒	☐	Clinical data
☒	☐	Dual use research of concern

Methods

n/a	Involved in the study	
☒	☐	ChIP-seq
☒	☐	Flow cytometry
☒	☐	MRI-based neuroimaging

Antibodies

Antibodies used	anti-TAP1 (clone mAb 148.3, dilution 1:20, produced in house)<br>anti-TAP2 (clone mAb 438.3, dilution 1:20, produced in house)<br>anti-tapasin (Abcam, dilution 1:1000, catalogue number ab13518)<br>anti-HLA-A/B/C HC10 (Acris Antibodies, dilution 1:1000, catalogue number AM33035PU-N)<br>anti-HLA-A (Abcam, dilution 1:1000, catalogue number ab52922)<br>anti-ERp57 (Abcam, dilution 1:2000, catalogue number ab10287)<br>anti-calreticulin (Sigma, dilution 1:1000, catalogue number C4606)<br>anti-β2m (Novo Antibodies, dilution 1:1000, catalogue number HPA006361)<br>anti-SBP antibody (Santa Cruz, dilution 1:500, catalogue number sc-101595)<br>anti-His6 antibody (Sigma, dilution 1:1000, catalogue number H1029)
Validation	anti-TAP1 (clone mAb 148.3) see Meyer TH et. al. (1994) FEBS lett DOI: 10.1016/0014-5793(94)00908-2, validated for Western blotting, human target<br><br>anti-TAP2 (clone mAb 438.3) see van Endert PM et. al. (1994) Immunity DOI: 10.1016/1074, validated for Western blotting, human target<br>anti-HLA-A/B/C HC10 (Acris Antibodies, catalogue number AM33035PU-N) has been thoroughly evaluated by us and others for Western blotting, human target:<br>1. Blees A et. al. (2017) Nature DOI: 10.1038/nature24627<br>2. Stam NJ et al. (1990) Int Immunol DOI: 10.1093/intimm/2.2.113<br>3. Gauster M et al. (2007) Rheumatology DOI: 10.1093/rheumatology/kel440<br><br>anti-HLA-A (Abcam, catalogue number ab52922) has been validated for Western blotting, human target by the manufacturer as stated on manufacturers website<br><br>anti-ERp57 (Abcam, human, Western blotting, catalogue number ab10287) has been validated for Western blotting, human target by the manufacturer as stated on manufacturers website<br><br>anti-calreticulin (Sigma, catalogue number C4606) has been validated for Western blotting, human target by the manufacturer as stated on manufacturers website<br><br>anti-β2m (Novo Antibodies, catalogue number HPA006361) has been validated for Western blotting, human target by the manufacturer as stated on manufacturers website<br><br>anti-SBP antibody (Santa Cruz, catalogue number sc-101595) has been validated for Western blotting, human target in Zhang et. al. (2022) Mol Cell DOI: 10.1016/j.molcel.2022.01.020<br><br>anti-His6 antibody (Sigma, catalogue number H1029) has been validated by the manufacturer as stated on manufacturers website

Eukaryotic cell lines

Policy information about cell lines

Cell line source(s)	Raji - Burkitt's lymphoma cells (Raji ATCC® CCL-86)
Authentication	Cell line is commerically available and was not authenticated.
Mycoplasma contamination	Cell lines were tested for mycoplasma contaminations
Commonly misidentified lines (See ICLAC register)	No commonly misidentified cell lines were used in this study
